# Supplementary material for: Differentiation-dependent proximity proteomics identifies novel host factors linked to HPV16 E2 function
Source: mBio. 2026 Jan 12;17(2):e03194-25. doi: 10.1128/mbio.03194-25 (PMC12892938; doi:10.1128/mbio.03194-25)
Supplement: Figure S1 — Western blot for C33a. [file mbio.03194-25-s0001.docx]

**
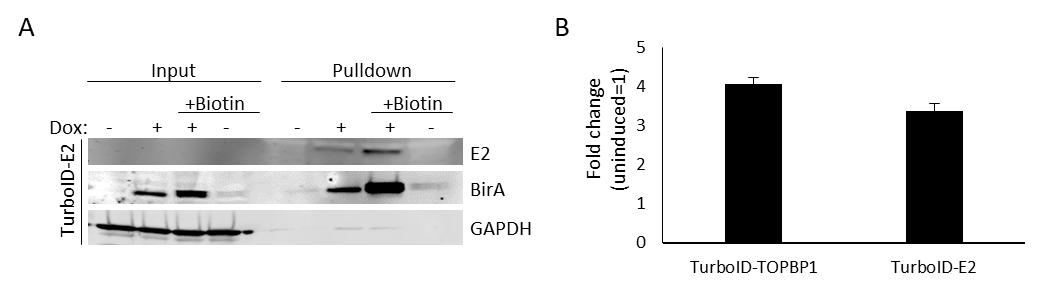
**

**Supplementary Figure 1: Generation of stable C33a lines expressing inducible doxycycline-inducible Turbo-ID tagged proteins.**

1. Western blot analysis confirmed inducible expression of TurboID-tagged E2 in C33a cells. Streptavidin-bead pulldown confirmed successful enrichment of the bait protein.
2. qRT-PCR using primers targeting the BirA confirmed induction of TurboID expression following doxycycline treatment. Fold changes were calculated relative to GAPDH and normalized to uninduced controls. Error bars represent standard deviation from biological replicates.
