# Supplementary material for: Differentiation-dependent proximity proteomics identifies novel host factors linked to HPV16 E2 function
Source: mBio. 2026 Jan 12;17(2):e03194-25. doi: 10.1128/mbio.03194-25 (PMC12892938; doi:10.1128/mbio.03194-25)
Supplement: Captions — for supplemental tables. [file mbio.03194-25-s0002.docx]

**Supplementary Table 1: HPV16 E2 and TOPBP1 interacting partners identified by TurboID proximity labeling.** This table lists the relative abundance of proteins identified by LC–MS/MS in N/Tert-1 keratinocytes expressing doxycycline-inducible TurboID-tagged HPV16 E2 or TOPBP1 under monolayer and differentiating (Ca²⁺-treated) conditions. And for N/Tert-1+HPV16 expressing doxycycline-inducible TurboID-tagged TOPBP1 under monolayer and differentiating conditions.

**Supplementary Table 2: Proteins identified in each condition for TurboID–E2 and TurboID–TOPBP1 proximity labeling.** This table provides condition-specific protein identifications from streptavidin-enriched lysates of TurboID tagged -E2 and -TOPBP1 keratinocytes. Separate sheets detail interactors detected under monolayer and differentiating (Ca²⁺-treated) conditions, including proteins unique to each condition and those shared between them. Previously reported E2 and TOPBP1 interactors are included for reference.
